# Latent Spinal Implant Infection During Pregnancy: A Case Report

**DOI:** 10.7759/cureus.78147

**Published:** 2025-01-28

**Authors:** Ian H Wong, Anas Bardeesi, Brett Rocos

**Affiliations:** 1 Johns Hopkins Bloomberg School of Public Health, Johns Hopkins University, Baltimore, USA; 2 Division of Spine Surgery, Duke University Hospital, Durham, USA

**Keywords:** best practices, multidisciplinary care team, pregnancy, spinal fusion, surgical site infections

## Abstract

Deep spinal surgical site infections (SSIs) following spine surgery pose a significant concern, with management becoming even more complex and high-risk in pregnant patients with spinal instrumentation. The optimal approach to managing this rare scenario remains unclear. We present the case of a 33-year-old pregnant woman who developed a delayed, deep spinal SSI several years after undergoing instrumented fusion for scoliosis. Due to her pregnancy, intervention was deferred until the postpartum period. A multidisciplinary team successfully managed the case with extensive debridement, removal of all instrumentation, and subsequent antibiotic therapy. This case highlights the importance of a multidisciplinary approach in managing complex SSIs during pregnancy. Given the limited data available, we advocate for individualized treatment decisions guided by a thorough risk-benefit analysis. Further research is needed to establish evidence-based guidelines for managing SSIs in this unique patient population.

## Introduction

Surgical site infections (SSIs) following spine surgery remain a significant concern, with reported incidence rates ranging from 2% to 20% [1,2]. Deep SSIs specifically occur at a rate of approximately 1.7% [3]. These infections typically manifest through back pain, purulent drainage, wound dehiscence, and soft tissue induration [4]. Various patient-related factors increase the risk of SSIs, including uncontrolled diabetes mellitus, tobacco use, obesity, prolonged corticosteroid therapy, and other systemic conditions that impair wound healing [4]. Additionally, procedural factors such as the posterior approach, implant placement, allograft utilization, and extended operative duration correlate with increased infection risk [5].

Although pregnancy was once considered an immunocompromised state, it is now understood that the immune system remains intact but is regulated to accommodate the fetus [6,7]. However, the independent association between pregnancy and spinal implant infection or failure remains unclear and has not yet been investigated. Deep SSIs in pregnant patients with spinal instrumentation pose significant challenges to both the patient and the treating team. Current surgical management strategies for these complex cases continue to evolve, particularly regarding the necessity of hardware removal during debridement procedures. These cases are further complicated by the need for coordinated care with the anesthesia and perinatology teams to ensure timely intervention without endangering the pregnancy.

This report discusses a delayed, deep spinal SSI that either developed or became symptomatic during pregnancy in a patient who had undergone posterior instrumented fusion for adolescent idiopathic scoliosis a decade prior. While previous reports have explored revision surgery during pregnancy for hardware failure and trauma, to the best of our knowledge, this is the first reported case addressing the management of a chronic deep SSI in the spine. The case emphasizes the importance of a multidisciplinary approach to ensure optimal outcomes for both the patient and the fetus.

## Case presentation

A 33-year-old female patient at eight weeks gestation presented with a two-month history of right lumbar and right lower extremity pain and paresthesia. She had previously been diagnosed with progressive pseudorheumatoid dysplasia and had undergone correction of syndromic scoliosis at age 14. This was followed by revision and extension of the fusion from T2 to L4 at age 27. Physical examination revealed prominence of the hardware at both cranial and caudal ends without external signs of inflammation. Both lower extremities demonstrated normal power on assessment, with pre-existing, stable, non-dermatomal sensory changes in the right leg. Physical therapy and symptomatic care were initiated as part of a conservative management plan, while radiological investigations were deferred due to pregnancy.

**Figure 1:**
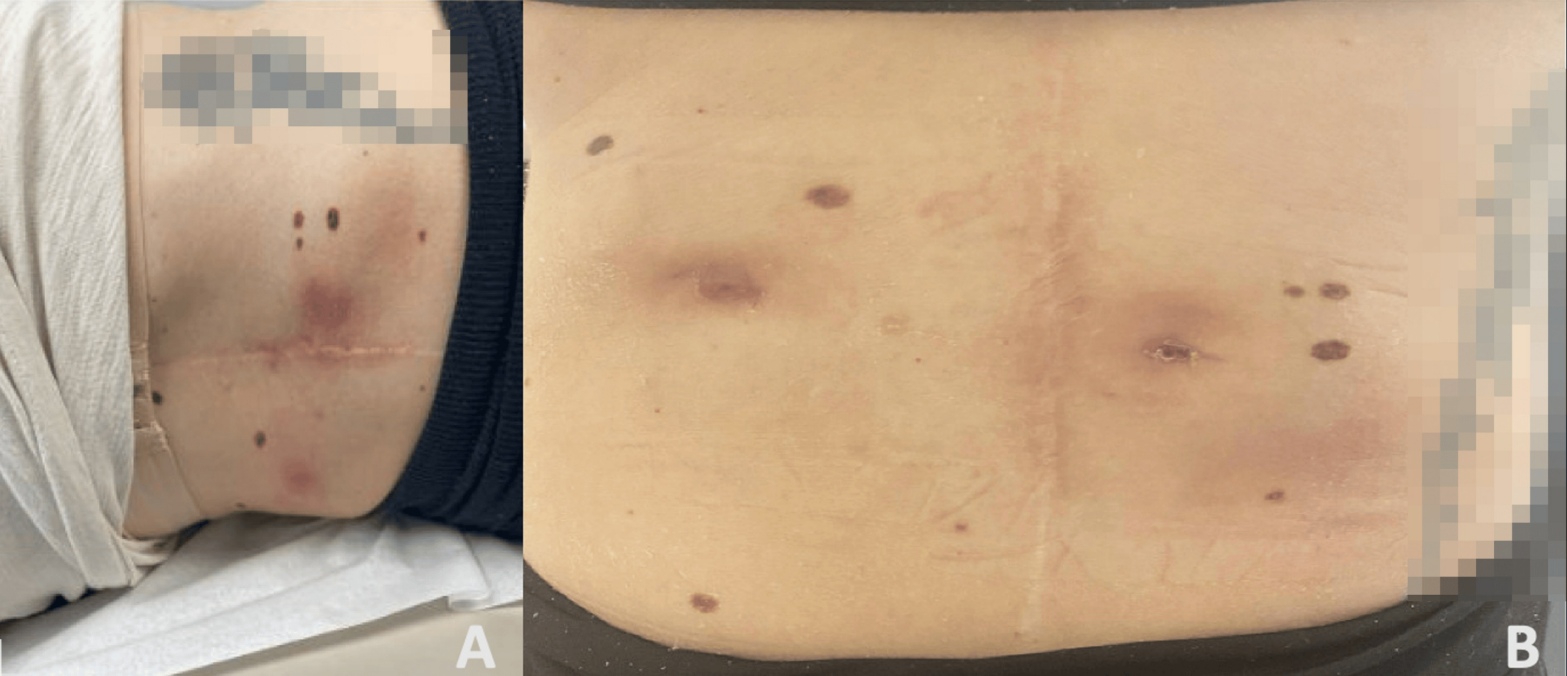
(A) Bilateral subcutaneous abscesses at 28 weeks gestation. (B) Bilateral sinuses that occasionally drained prior to delivery date.

**Figure 2:**
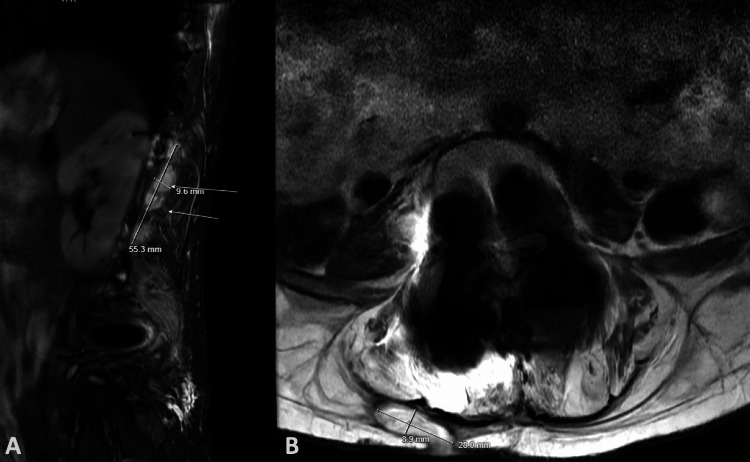
(A) Sagittal T2-weighted MRI showing a left-sided T11-L2 multilocular fluid collection lateral to the hardware and (B) axial cut of the same study showing a right-sided paraspinal muscle focal fluid collection spanning the L3-5 levels with a draining sinus.

At 28 weeks gestation, the patient’s right-sided lumbar pain worsened, accompanied by localized tenderness, edema, and a rash (Figure 1). Dermatology consultation identified two suspicious subcutaneous nodules suggestive of underlying abscess formation. Ultrasound and MRI revealed two fluid collections on either side of the lumbar spine (5.5 cm^3^ right, 3.5 cm^3^ left), both of which were drained and confirmed to be abscesses (Figure 2). Oral linezolid was started empirically but discontinued after 48 hours of treatment when Gram stains and cultures showed no evidence of infection.

The multidisciplinary team, comprising spine surgery, obstetrics, and infectious diseases specialists, considered chronic implant infection as a differential diagnosis. Given the absence of systemic infection and if the patient maintained an uneventful pregnancy, the consensus was to postpone surgical exploration and debridement until after delivery. The patient remained clinically stable with manageable intermittent lumbar pain. At 30 weeks gestation, two sinuses erupted over the lumbar instrumentation, draining intermittently without cutaneous erythema or contained collection, both managed with routine dressing changes. Laboratory studies revealed normal leukocyte count but slightly elevated inflammatory markers (C-reactive protein (CRP) level 4.5 mg/dL, erythrocyte sedimentation rate (ESR) 80 mm/hour). The patient was reviewed biweekly by the obstetric service. A planned cesarean delivery was carried out at 37 weeks gestation resulting in the birth of a healthy neonate with no complications.

**Figure 3:**
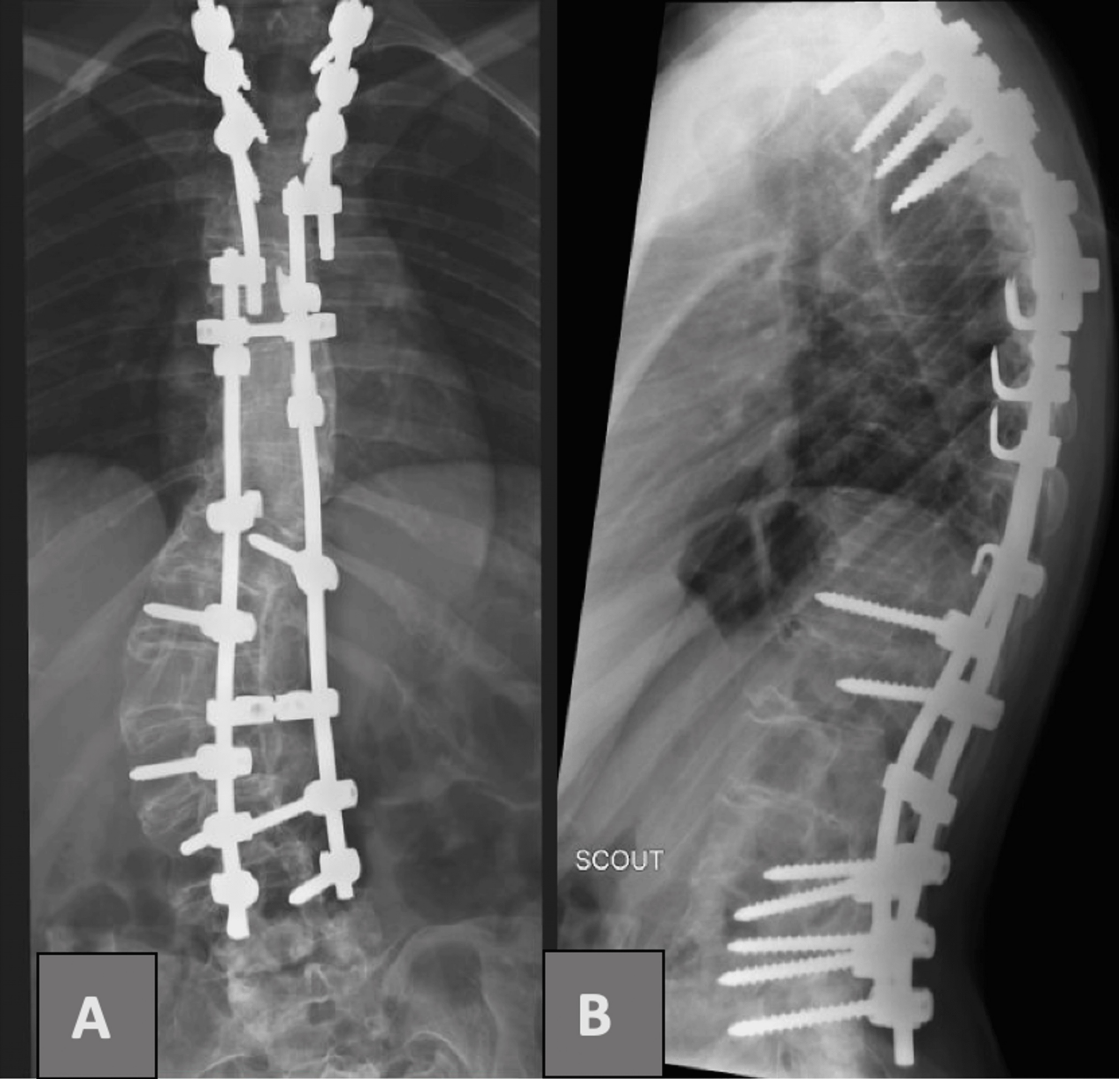
(A) Anteroposterior and (B) lateral views of the hardware from T2 to L4 levels.

Surgical debridement and exploration were performed on postpartum day five. The previous incision was reopened, multiple tissue samples at various depths were obtained for analysis, and the wound was thoroughly debrided and irrigated. Gross infection was noted along the length of the instrumentation, which was removed in its entirety. Loosening of the bilateral L4 pedicle screws was also observed (Figure 3). Multiple infected soft tissue pockets and screw holes were debrided. The plastic surgery team then performed myofascial flap reconstruction with multilayer closure and negative pressure wound therapy application.

The postoperative course was favorable, with the patient remaining clinically stable and reporting gradual but significant improvement in lumbar pain. Tissue cultures grew *Propionibacterium acnes*, which was treated with a six-week course of daily intravenous ceftriaxone through a peripherally inserted central catheter. The patient completed the antibiotic course without complications, demonstrating normalization of inflammatory markers (CRP level 0.13 mg/dL, ESR 6 mm/hour). At the six-week follow-up, the patient showed satisfactory recovery. During this evaluation, no concerns were identified regarding fetal health or development. The patient opted for further follow-up on an as-needed basis.

## Discussion

Management of spinal SSIs in the presence of instrumentation is challenging due to the lack of high-quality, evidence-based guidelines. Current management algorithms, proposed from case studies and expert opinions, encompass various approaches: debridement with hardware retention, hardware exchange, or complete implant removal, each combined with appropriate antibiotic therapy [8-11]. Treatment selection depends primarily on patient-specific factors, including operability, SSI onset timing, spinal stability, and microbial complexity [9].

Although the definition of early infection varies among studies (< 6 weeks versus < 3 months) [8,9], management recommendations generally involve retention of instrumentation with removal of any loose implants. Late infections may warrant complete hardware removal or exchange, performed either as single- or two-stage procedures. Bürger et al. proposed a management algorithm considering the onset of the SSI and microbial virulence [9]. Their protocol suggests antibiotic therapy for either eradication or long-term suppression based on antimicrobial resistance and biofilm-forming potential. Decisions regarding complete instrumentation removal consider the patient's underlying condition, spinal stability after removal, and risk of neurological deterioration.

Given the paucity of literature, deep SSIs in gravid patients must be approached on an individualized basis, carefully balancing maternal and fetal risks. Non-obstetric surgery during pregnancy occurs infrequently, with reported rates of approximately 1-2% [12], predominantly for acute abdomen. While studies demonstrate an increased incidence of very low birth weight infants, they show no elevated risk of stillbirth or congenital anomalies [12]. Although no fetal complications were reported among 34 pregnant patients undergoing spine surgery in these series [12-14], this limited sample size precludes definitive conclusions regarding the safety of surgical positioning and anesthesia.

Spinal infections in the setting of pregnancy remain rare, with reported cases primarily following peripartum interventions (e.g., epidural anesthesia) or occurring in immunocompromised states such as uncontrolled diabetes mellitus or intravenous drug use [15]. Cases of spontaneous infection have also been documented [7]. Previously, pregnancy was viewed as a period of immunosuppression, leaving pregnant people more susceptible to infections [6]. However, recent efforts by Mor et al. suggest otherwise, recognizing pregnancy as a triphasic immunological process, characterized by pro-inflammatory first and third trimesters separated by an anti-inflammatory second trimester [6]. This immunological modulation may confer protection against infection, even in the presence of interventions or comorbidities.

In this case, our patient presented during the first trimester with lumbar pain. With no definitive signs of underlying infection and based on her clinical presentation, we initially suspected the need for deformity correction and fusion extension. However, we recommended postponing surgery until the postpartum period. The subsequent development of subcutaneous abscesses in the second trimester, without neurological compromise or a systemic inflammatory response, introduced unique management challenges. Most existing literature on spine surgery during pregnancy primarily addresses cases involving cord compression, cervical epidural abscesses, or paraplegia [7,16]. Butenschoen et al. proposed an algorithm for managing spine surgery in pregnancy, focusing on gestational age, positioning, and anesthetic considerations, but without accounting for the complexity of individual cases [12]. Their series primarily dealt with emergency disc herniations, which comprised two-thirds of the cases, with a median operative time of 90 minutes. In contrast, our case involved a significantly longer operative time of approximately 300 minutes and considerable blood loss (1500 mL), which could have posed further risks to both the patient and the pregnancy had surgery been performed prenatally. Consequently, we advocate for a case-by-case, multidisciplinary approach when managing spinal conditions during pregnancy, particularly in complex cases requiring extensive washout, debridement, and potential hardware exchange or removal. This approach ensures that treatment decisions are tailored to the needs of both the mother and the fetus.

## Conclusions

The successful management of this complex case of deep spinal SSI during pregnancy was made possible through the coordinated efforts of a multidisciplinary team. However, the scarcity of such cases, combined with the limited evidence available in the literature, highlights the challenges of managing pregnant patients with spinal implant infections. Developing structured, evidence-based approaches to treat these rare yet significant infections is crucial for optimizing outcomes in this patient population.
